# Effect of room temperature on tests for diagnosing vibration-induced white finger: finger rewarming times and finger systolic blood pressures

**DOI:** 10.1007/s00420-017-1214-2

**Published:** 2017-03-28

**Authors:** Ying Ye, Michael J. Griffin

**Affiliations:** Human Factors Research Unit, Institute of Sound and Vibration Research, University of Southampton, Southampton, SO17 1BJ UK

**Keywords:** Vibration-induced white finger, Finger skin temperature, Hand-arm vibration syndrome, Hand-transmitted vibration, Finger systolic blood pressure, Cold provocation test

## Abstract

**Purpose:**

This study investigates the effects of room temperature on two standard tests used to assist the diagnosis of vibration-induced white finger (VWF): finger rewarming times and finger systolic blood pressures.

**Methods:**

Twelve healthy males and twelve healthy females participated in four sessions to obtain either finger skin temperatures (FSTs) during cooling and rewarming of the hand or finger systolic blood pressures (FSBPs) after local cooling of the fingers to 15 and 10 °C. The measures were obtained with the room temperature at either 20 or 28 °C.

**Results:**

There were lower baseline finger skin temperatures, longer finger rewarming times, and lower finger systolic blood pressures with the room temperature at 20 than 28 °C. However, percentage reductions in FSBP at 15 and 10 °C relative to 30 °C (i.e. %FSBP) did not differ between the two room temperatures. Females had lower baseline FSTs, longer rewarming times, and lower FSBPs than males, but %FSBPs were similar in males and females.

**Conclusions:**

Finger rewarming times after cold provocation are heavily influenced by room temperature and gender. For evaluating peripheral circulatory function using finger rewarming times, the room temperature must be strictly controlled, and a different diagnostic criterion is required for females. The calculation of percentage changes in finger systolic blood pressure at 15 and 10 °C relative to 30 °C reduces effects of both room temperature and gender, and the test may be used in conditions where the ±1 °C tolerance on room temperature required by the current standard cannot be achieved.

## Introduction

Workers who are regularly exposed to hand-transmitted vibration from powered hand tools are at risk of developing disorders in the fingers, hands or arms, collectively known as the hand-arm vibration syndrome (Griffin 1997; Griffin and Bovenzi 2002). One consequence of exposure to hand-transmitted vibration can be impaired circulation in the fingers, with ‘attacks’ of finger blanching provoked by exposure to cold. The blanching may occur on the distal, middle, or proximal phalanges of the fingers and is called ‘vibration-induced white finger’, VWF, sometimes considered a form of secondary Raynaud’s disease (Griffin 1990).

The diagnosis of vibration-induced white finger is currently heavily reliant on the reporting of relevant symptoms, such as cold-induced finger blanching, and an appropriate history of exposure to hand-transmitted vibration. Cold provocation of the fingers and hands is commonly used in clinical and epidemiological studies to seek confirmation of the existence of an abnormal response to cold in the digital vessels of workers reporting relevant symptoms. Two vascular tests involving exposure to cold have been standardised: the measurement of finger rewarming times after cold provocation (ISO 14835-1:2016) and the measurement of finger systolic blood pressures during cold provocation (ISO 14835-2:2005).

The measurement of finger rewarming times involves immersion of the hands in cold water for a period of time and then the recording of the recovery of finger skin temperature. The diagnostic power of the test is uncertain, because there have been wide variations in both the nature of the cold challenge and the diagnostic criteria used to identify abnormality (e.g. Hack et al. 1986; Kurozawa et al. 1991; Virokannas and Rintamäki 1991; Lawson and Nevell 1997; Harada 2002; Cherniack et al. 2003). It has been suggested the test may be useful for discriminating on a group basis between those with and those without vibration-induced white finger, but that it may lack the sensitivity and specificity to distinguish between individuals with and without vibration-induced white finger (Bogadi-Sare and Zavalic 1994; Lawson and Nevell 1997). This has led to the diagnosis of vibration-induced white finger very often being based solely on the reported symptoms without any confirmation of the signs of disorder. Finger skin temperature in air depends on the environmental conditions as well as blood flow through the digit (Bovenzi 1987). It might be expected that the repeatability of the test and its reliability as an indicator of digital vasospasm in workers with VWF will depend on the environmental conditions before, during, and after the hands are immersed in water.

The measurement of finger systolic blood pressures after cold provocation is considered a promising laboratory test for quantifying the degree of cold-induced digital vasospasm in vibration-exposed workers (Thulesius et al. 1981; Olsen et al. 1995; Gemne 1997; Bovenzi 2002; Bovenzi et al. 2008). The results of the test are usually expressed in terms of percentage FSBP (%FSBP), which is the systolic blood pressure in a test finger cooled to 15, 10, or 6 °C expressed as a percentage of the systolic blood pressure at 30 °C, corrected for the change of pressure in an ipsilateral non-cooled reference finger during the cold test (Ekenvall and Lindblad 1986; Bovenzi 1988, 1993). During cold provocation of the fingers, the blood vessels constrict and finger systolic blood pressure falls (Nielsen and Lassen 1977; Nielsen et al. 1980). The cold-induced reductions in finger systolic blood pressures seem to be related to reports of finger blanching with high repeatability, sensitivity, and specificity (e.g. Hack et al. 1986; Kurozawa et al. 1991; Carnicelli et al. 1992; Nasu and Kurozawa 1995; Gemne 1997; Ye and Griffin 2016).

A study of FST after immersion of one hand in cold water at 10 °C for 10 min with room temperatures from 10 to 30 °C has suggested that FST is strongly affected by room temperature (Harada et al. 1998). Published studies of FSBP after cold provocation have been conducted with various room temperatures (16–26 °C) in healthy control groups, in workers without symptoms but exposed to vibration, and in VWF patients (Kurozawa et al. 1991; Ekenvall and Lindblad 1986; Harada et al. 1998; Bovenzi 1993). In ISO 14835 (2005), the room temperature for the FST and FSBP tests is set at 21 ± 1 °C for the duration of the test and the air circulation must be controlled to avoid skin cooling. Although environmental temperature influences peripheral circulation (Harada et al. 1998; Mirbod et al. 1998; Ye and Griffin 2011a), there is no study designed to understand the relationship between room temperature and finger systolic blood pressures after local cooling.

Lower baseline finger blood flow and lower FST have been reported in females (Cooke et al. 1990; Ye and Griffin 2011a). Studies have found higher blood pressures in males than in females of similar age (Wiinber et al. 1995; Khoury et al. 1992; Reckelhoff 2001). The gender-associated differences in blood pressures observed in humans have also been reported in some animal models (Masubuchi et al. 1982; Chen and Meng 1991).

This study investigated: (1) how room temperature influences changes in finger skin temperatures and finger systolic blood pressures after cold provocation in the digits of healthy males and females and (2) gender differences in finger skin temperature and finger systolic blood pressures after cold provocation. The study was undertaken at two room temperatures: 20 and 28 °C. The tests were performed in the same conditions by an experimenter experienced in applying the tests according to the HSE recommended procedure (Lindsell and Griffin 1998). It was hypothesised that there would be shorter recovery period for finger skin temperature after cold provocation and higher finger systolic blood pressures after digital cooling at 15 and 10 °C when the tests were conducted in the higher room temperature. For the rewarming test, it was hypothesised that males would have a higher FST before cooling and shorter recovery periods after cooling than females. For the FSBP test, it was hypothesised that males would have higher FSBPs than females both before and after the cold provocation.

## Method

### Subjects

Twenty-four healthy volunteers, 12 males and 12 females, participated in the study. All subjects were university students with no history of significant (regular or prolonged) exposure to hand-transmitted vibration in occupational or leisure activities. They completed a health questionnaire, read a list of medical contraindications, and gave their written informed consent to the study. None of the subjects reported cardiovascular or neurological disorders, connective tissue diseases, injuries to the upper extremities, or a family history of Raynaud’s phenomenon. All were right-handed and non-smokers.

The subjects were requested to avoid consuming caffeine for 4 h and alcohol for 12 h prior to the testing. The study was approved by the Ethics Committee of the Faculty of Engineering and the Environment (Application number: 12795).

### Finger skin temperatures

An *HVLab* 8-channel temperature monitor (University of Southampton) was used to measure the finger skin temperature following cold provocation in accord with ISO 14835-2:2016. Calibrated thermocouples were attached to the fingertips of the thumb, index, ring, and little fingers, and the distal, median, and proximal phalanges of the middle finger on the right hand, which was gloved in thin plastic so as to remain dry during the cold challenge. One thermocouple was attached to the fingertip of the left middle finger as a reference.

During the test, subjects sat on a seat next to a table supporting a water bath. The height of the seat was adjusted so that subjects were comfortable and able to maintain a similar posture throughout both the cooling period (i.e. during immersion) and the subsequent rewarming period. After a settling period of 2 min with both hands at heart level, the right hand was immersed in stirred water at 15 °C for 5 min. The subject then gently removed the right hand from the water with the help with the experimenter, the thin glove was removed, and the right hand kept at heart level to rewarm for 40 min. During the test, the left hand rested on a foam support at heart level and remained motionless. There was continuous monitoring of skin temperature during the pre-immersion, the hand cooling, and the rewarming period using a computer and *HVLab* diagnostic software (version 8.5, University of Southampton). A graph was subsequently produced showing changes in the temperatures of the eight thermocouples over time. The time for FST to increase by 4 °C (*T*_4_) after cold provocation and the time for FST to return to within 2 °C of the baseline finger temperature (*T*_2-base_) were used as criteria to indicate the finger rewarming times (Carnicelli et al. 1992; Lindsell and Griffin 1998).

### Finger systolic blood pressures

An *HVLab* plethysmograph (University of Southampton) was used to measure finger systolic blood pressures following cold provocation of the digits in accord with ISO 14835-1:2005. Water-perfusable cuffs were placed around the middle phalanx of each finger, with a separate air cuff around the thumb as a reference. Strain gauges were placed at the base of the finger nails of the cuffed fingers. Subjects lay supine and motionless on a couch with both hands resting in a comfortable position at the level of the heart so as to minimise effects of hydrostatic variations. The tips of the fingers were squeezed to reduce blood volume and then the cuffs were inflated to 220 mm Hg (a suprasystolic pressure to prevent arterial inflow) by perfusing the cuffs with thermostatically controlled water. After 5 min of ischaemia, the cuff pressure was reduced at a rate of 2 mm Hg/s. Finger systolic blood pressures were measured on the right hand after cooling by water circulating at 30, 15, and 10 °C. The finger systolic blood pressure was calculated as the cuff inflation pressure at which arterial inflow returned to the finger at 30, 15, and 10 °C. The percentage changes in finger systolic blood pressure (%FSBP) from 30 to 15 °C, and from 30 to 10 °C, were calculated according to the following equation:

$$\% FSBP_{{t^\circ {\text{C}}}} = \frac{{FSBP_{{{\text{test,}}t^\circ {\text{C}}}} }}{{FSBP_{{\text{test,30}}^\circ {\text{C}}} - (FSBP_{{\text{ref,30}}^\circ {\text{C}}} - FSBP_{{\text{ref,}}t^\circ {\text{C}}})}} \times 100\%$$

where FSBP_t°C_ is the finger systolic pressure of the test finger after thermal provocation at 10 or 15 °C; FSBP_test,30 °C_ is the finger systolic blood pressure measured on the test digit after thermal provocation at 30 °C; FSBP_ref,30 °C_ is the finger systolic blood pressure measured on the reference finger after thermal provocation of the test finger at 30 °C; and FSBP_ref,*t*°C_ is the finger systolic blood pressure measured on the reference finger after thermal provocation of the test finger at 10 or 15 °C.

Finger skin temperature (FST) was measured using *k* type thermocouples attached by micro pore tape to the distal phalanges of the right and left middle fingers during the FSBP measurements.

### Room temperature

The temperature of the room was controlled by air conditioning. The air flow was not noticeable.

The room temperature was measured by a mercury-in-glass thermometer to an accuracy of ±0.5 °C. The thermometer was located close to the heads of the subjects.

### Procedure

Initially, subjects stayed in one of the two room temperatures (either 20 or 28 °C) for at least 30 min or until they had a constant finger skin temperature (<1 °C variation over 10 min) before the test started. Each subject participated in four sessions conducted on four separate days during the winter months (in November and December). In each session, either the finger systolic blood pressures at three water temperatures (starting with 30 °C, then 15 °C, and finally 10 °C) or the finger skin temperatures on the right hand were measured with room temperature at either 20 or 28 °C. The order of presentation of the four conditions was randomised.

During the two FSBP measurement sessions, the FST on the right and left middle fingers were measured before and after the FSBP measurements at the three water temperatures (30, 15, and 10 °C).

### Statistical methods

Data analysis was performed using the software package SPSS (version 22.0). The data were summarised with the median as a measure of central tendency and the interquartile range (IQR) as a measure of dispersion. Non-parametric tests were employed to analyse the data, which were not normally distributed. The Wilcoxon test was used to investigate differences between the measures of (1) FST before immersion, at the last minute of the immersion period, the time to rewarm by 4 °C, and the time for FST to return within 2 °C of the baseline temperature with each of the two room temperatures (i.e. 20 or 28 °C); (2) FSBP and %FSBP with the two room temperatures and the two water temperatures (i.e. 15 and 10 °C). The Friedman test was used to investigate differences between measurement locations for FST, FSBP and %FSBP. The Mann–Whitney *U* test was used to investigate differences between males and females. The Spearman rank correlation coefficient was used to investigate associations between FSTs, finger rewarming times, FSBPs, and individual body and finger sizes.

The criterion for statistical significance was *p* < 0.05. The reported *p* values have been adjusted for multiple comparisons.

## Results

The medians and IQRs of the age and body size of the male and female subjects are shown in Table 1. There were significant differences between male and female subjects in their stature (*p* = 0.008), weight (*p* < 0.001), finger volume (*p* < 0.01), and body mass index (BMI) (*p* < 0.001). The male and female subgroups did not differ in age (*p* = 0.36).

**Table 1:** Median and interquartile range (IQR) of the age, body size, and finger size for male and female subjects

	Females	Males
Age (years)	24 (21–27)	23.5 (20–26)
Height (m)	1.61 (1.56–1.66)	1.76 (1.72–1.80)
Weight (kg)	53 (47–61)	75 (60–87)
Finger volume (cm³)		
Right middle	12.5 (11.2–13.6)	15.4 (12.9–17.4)
Left middle	12.3 (11.0–13.8)	15.0 (13.3–17.1)
Body mass index (BMI)	20.5 (19.7–21.8)	23.1 (21.5–24.8)

In the four sessions, the temperature in the laboratory was either in the range of 19.5–21.0 °C or in the range of 27.0–28.5 °C. Within the four sessions, the room temperature did not change significantly during any session (at 20 °C, *p* = 0.346–0.641; at 28 °C, *p* = 0.237*–*0.512).

### Finger skin temperature after cold provocation

For temperatures measured on the distal phalanges (i.e. fingertips) of the right hand, the medians and interquartile ranges of temperatures during the 2-min baseline, at the end of the 5th minute of cooling, the times to rewarm by 4 °C and the times for FST to return within 2 °C of baseline temperature in males and females are shown in Table 2.

**Table 2:** Medians and interquartile ranges (IQR) of finger skin temperature (FST), the time to increase FST by 4 °C (*T*_4_), and the time for FST to return within 2 °C of initial temperature (*T*_2-base_) on the distal phalanges of the right hand in 12 males and 12 females

Fingers (right hand)		Room temperature of 20 °C						Room temperature of 28 °C					
		Thumb	Index	Middle	Ring	Little	Reference finger	Thumb	Index	Middle	Ring	Little	Reference finger
Baseline FST (°C)	Male	33.9* (32.2–34.5)	32.9* (31.8–34.8)	33.1* (32.0–35.3)	32.6* (31.9–34.4)	32.4* (31.8–34.5)	33.0* (32.0–34.2)	35.1 (34.1–35.6)	35.1 (33.9–36.0)	35.0 (34.0–35.9)	34.8 (33.8–36.4)	35.0 (34.1–36.7)	35.1 (33.6–36.1)
	Female	31.9* (26.2–33.1)	31.9* (26.4–33.8)	31.5* (26.0–34.3)	31.0* (24.9–33.9)	30.9* (24.8–33.5)	31.2* (26.1–33.3)	33.7 (33.1– 34.5)	33.3 (33.0–34.1)	33.2 (32.5–34.1)	33.4 (32.8–34.4)	33.0 (31.7–33.9)	33.2 (32.9–34.0)
FST at the 5th min of immersion (°C)	Male	16.1* (15.8–17.1)	15.7* (15.5–16.8)	16.1* (15.0–16.9)	15.6* (15.0– 15.8)	15.9* (15.4–16.5)	32.0* (31.1–33.6)	17.0 (16.5–17.3)	17.1 (16.4–17.9)	17.1 (16.7–18.3)	16.5 (15.9–16.8)	16.5 (15.4–17.6)	33.7 (32.0–35.4)
	Female	15.6 (15.2–16.1)	15.3 (15.0–15.7)	15.1 (15.0–15.4)	15.5 (14.9–15.8)	15.2 (14.9–15.3)	30.4* (25.7–32.2)	15.7 (15.0–16.3)	15.6 (15.4–15.9)	16.0 (15.7–16.5)	15.5 (15.0–16.2)	15.5 (15.1–15.9)	32.1 (31.3–33.0)
*T*_4_ (s)	Male	135** (102–153)	144** (105–176)	144** (110–167)	145** (97–163)	148** (101–178)	N/A	95 (62–123)	105 (91–134)	103 (90–137)	105 (87–151)	110 (90–157)	N/A
	Female	175** (142–213)	184** (145–244)	183** (149–257)	185** (146–263)	188** (150–287)	N/A	123 (91–153)	134 (99–164)	133 (102–167)	135 (110–153)	138 (103–177)	N/A
*T*_2-base_ (min)	Male	19.2** (15.2–25.5)	21.3** (15.0–26.6)	21.2** (16.1–25.8)	22.1** (16.2–26.7)	22.3** (15.9–26.5)	N/A	14.7 (10.2–18.3)	16.3 (11.5–19.4)	16.0 (12.9–21.7)	15.8 (10.7–21.3)	15.7 (12.0–21.7)	N/A
	Female	20.4** (16.1–25.6)	21.8** (16.3–25.5)	21.6** (16.5–26.2)	22.2** (17.1–26.0)	23.0** (17.4–26.8)	N/A	16.5 (13.2–19.8)	17.1 (15.5–23.4)	17.8 (14.9–22.7)	17.6 (14.7–23.0)	17.3 (15.0–22.7)	N/A

*Left middle finger* used as a reference for finger rewarming

*p < 0.01, **p < 0.001: significant lower FST at baseline and at the 5th minute of immersion period and significant increase in rewarming times with room temperature of 20 °C (Wilcoxon test)

**Fig. 1:**
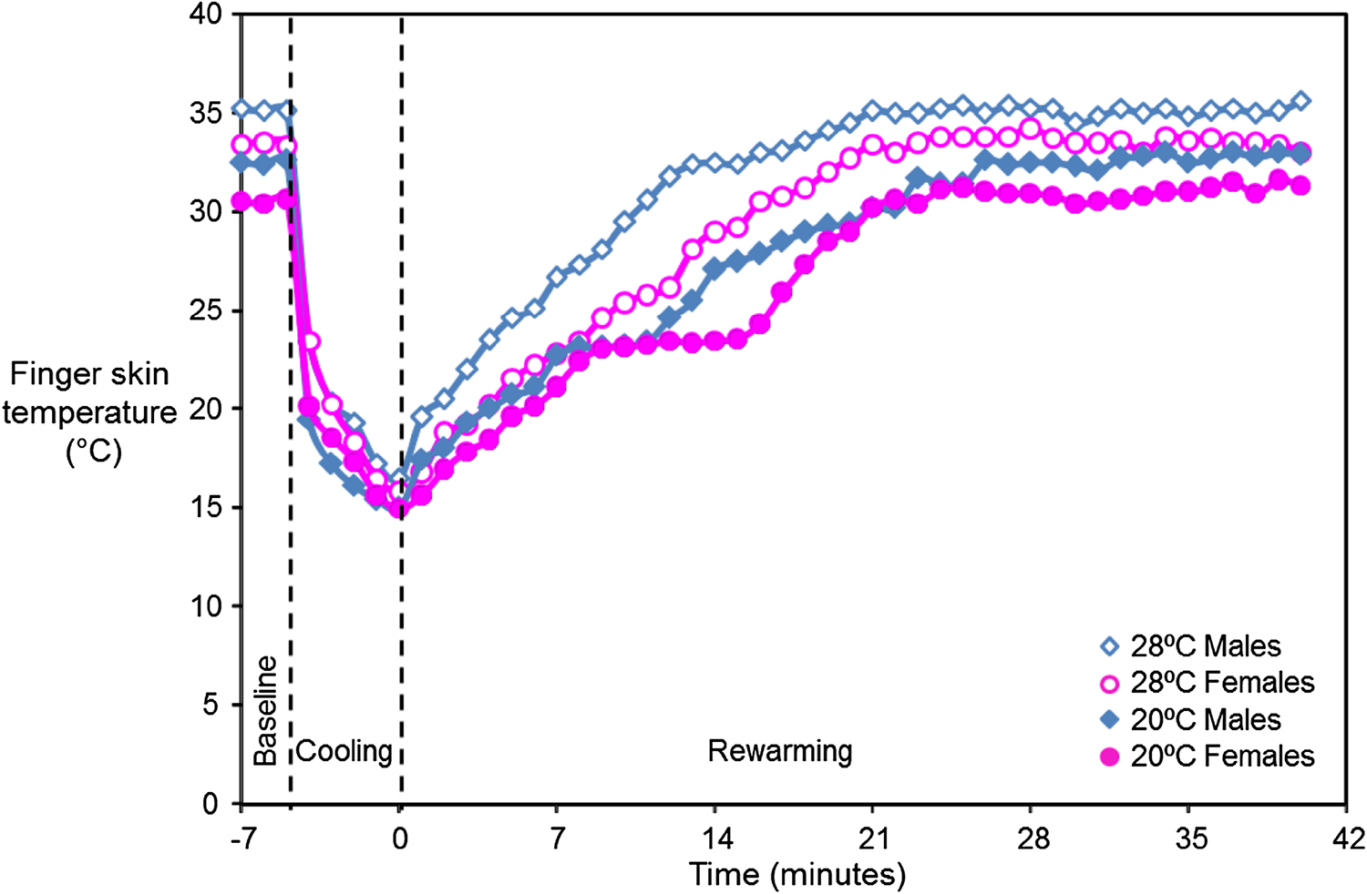
Median finger skin temperatures before, during, and after immersion of the hand in stirred water at 15 °C for 5 min. Data from 24 subjects

Median FST on the distal phalanx of the right hand before, during, and after cold immersion with room temperatures of 20 and 28 °C are shown for males and females in Fig. 1.

During the 2-min baseline, the skin temperatures on the distal phalanges of the thumbs, and the index, middle, ring and little fingers were significantly lower with the room temperature at 20 °C than at 28 °C for both males and females (*p* < 0.01). At both room temperatures, the FSTs during this period were lower in females than in males (*p* < 0.001). There were no significant differences in finger temperatures across different fingers in either males or females with either room temperature (*p* > 0.05), except for a lower skin temperature on the distal phalanges of the ring and little fingers in females at 20 °C (*p* < 0.01).

During the 5-minute of the cooling, on the right hand, there was a trend for slightly lower finger skin temperatures at the 5th minute of cold provocation with the room temperature at 20 °C, although statistically significant only in males (*p* < 0.01). There was a greater reduction in FSTs with the higher room temperature in both males and females (*p* < 0.001). The reduction in FST was greater in men than in women (*p* < 0.05).

During the first 8 min of the rewarming, the median FST increased at 3.2 °C/min in males and 2.0 °C/min in females with the 28 °C room temperature, but at only 1.8 °C in males and 1.7 °C/min in females with the 20 °C room temperature. Subsequently, the median FSTs show steady rewarming with the 28 °C room temperature, but with the room temperature at 20 °C, the rate of increase in median FST decreased to 0.1 °C/min in males over a 3-min period and in females over a 7-min period. Thereafter, the rate of increase in median FST remained at 1.0–1.2 °C/min until the median FST recovered to the initial temperature.

The time for FST to increase by 4 °C and the time for individual FSTs to return to within 2 °C of the baseline finger temperature was shorter with the higher room temperature in both males and females (*p* < 0.01), and shorter in males than in females at both room temperatures (*p* < 0.001).

On the middle finger of the left hand (i.e. reference finger not immersed in the cold water), there was a decrease in median FST during the immersion period compared to the 2-minute baseline with the room temperature at both 20 and 28 °C (*p* = 0.007–0.029). During the first 8 min of the rewarming period, there was a significant increase in FST compared to the FST at the 5th minute of cooling at both 20 and 28 °C in both males and females (*p* < 0.01). After return to the baseline temperature, there was no significant change in FST over the remaining period of recovery (*p* > 0.05).

The baseline FSTs within the male and female subgroups were not correlated with stature, weight, BMI, or age (*p* = 0.12–0.63). With both room temperatures, there was a positive correlation between the baseline FST and finger volume in both males (*p* = 0.006–0.024; Spearman) and females (*p* = 0.001–0.033).

### Finger systolic blood pressures after digital cooling test

#### Finger skin temperature during the measurement of FSBP

**Fig. 2:**
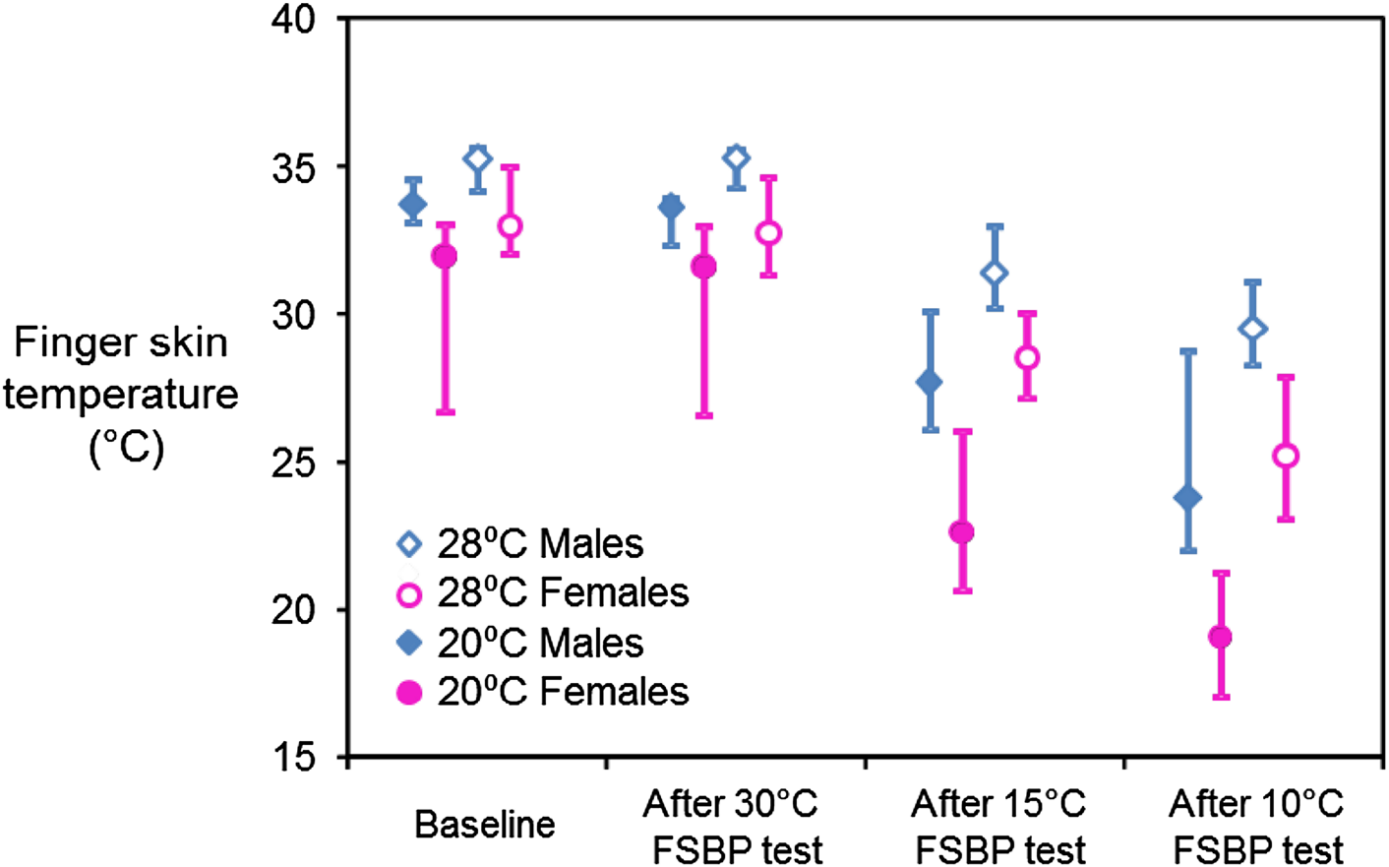
Medians and interquartile ranges (IQR) of finger skin temperature (FST) on the r*ight middle finger* before and after FSBP tests at 30, 15, and 10 °C water temperature with room temperatures of 20 and 28 °C. Data from 12 males and 12 females. Data from 24 subjects

At all four measurement times, the median FST was lower with the room temperature at 20 °C than at 28 °C, for both male and female subjects (Fig. 2).

Before the thermal provocation, the baseline finger skin temperature differed between male and female subjects as shown in Fig. 2. The FSTs on the right and left middle fingers did not differ between the baseline and after the 30 °C FSBP test with either of the two room temperatures (20 and 28 °C) in either male or female subjects (*p* = 0.166*–*0.604).

There was a significant reduction in finger skin temperature on the right middle finger after the FSBP test conducted at 15 and 10 °C compared with baseline and after 30 °C FSBP test with both room temperatures in both males and females (20 °C, *p* = 0.003–0.008; 28 °C, *p* < 0.001). On the left middle finger, there was a reduction in FST after the FSBP test at both 15 and 10 °C with both room temperatures in both males and females (*p* = 0.019–0.027), except with the 28 °C room temperature in males (*p* = 0.056). The extent of the reduction in FST was greater on the right middle finger exposed to cold than on the unexposed left middle finger (*p* < 0.001).

With both room temperatures during all FSBP measurements, the median finger skin temperatures on the right and left middle fingers were lower in females than males (baseline, after FSBP test at 30, 15, 10 °C) (*p* < 0.001).

#### Finger systolic blood pressure and %FSBP

**Fig. 3:**
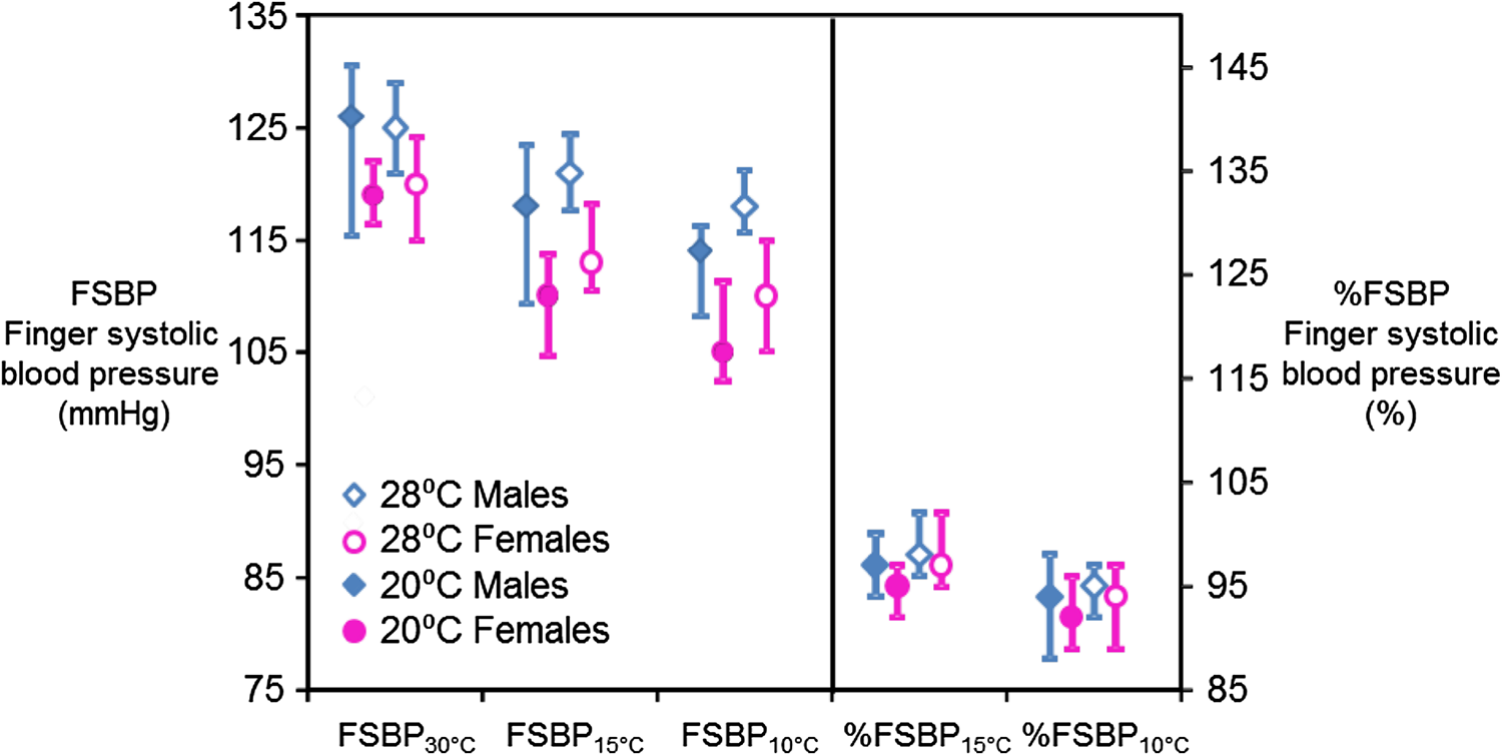
Medians and interquartile ranges (IQR) of finger systolic blood pressure (FSBP) with 30, 15, and 10 °C water temperatures and %FSBP calculated with 15 and 10 °C water temperatures and room temperatures of 20 and 28 °C. Data from 12 males and 12 females. Data from 24 subjects

The median and interquartile ranges of FSBPs measured at 30, 15, and 10 °C, and the %FSBP calculated for 15 and 10 °C with room temperatures of 20 and 28 °C for males and females are shown in Fig. 3.

At neither room temperature did the FSBP measured in air on the reference right thumb differ when the temperature of the other fingers was varied to 30, 15, or 10 °C, in either males or females (*p* = 0.56–0.61).

At neither room temperature did the FSBP obtained at 30, 15, or 10 °C differ across the index, middle, ring, and little fingers in either males or females (*p* = 0.29–0.56), except on the little finger where there was a lower FSBP at 10 °C with the room temperature of 20 °C in females (*p* = 0.013–0.047).

The FSBPs at 30 °C did not differ between the 20 and 28 °C room temperatures in either males or females (*p* = 0.19–0.33). However, FSBPs at 15 °C were lower with the room temperature at 20 °C than at 28 °C (*p* = 0.017–0.024) and FSBPs at 10 °C were also lower with the room temperature at 20 °C than at 28 °C (*p* = 0.001–0.008), in both males and females.

Notwithstanding the reductions in FSBPs, the %FSBPs calculated at 15 and 10 °C did not differ between room temperatures of 20 and 28 °C in either males or females (*p* = 0.081–0.28; Fig. 3).

The %FSBPs obtained at 10 °C were generally lower than those obtained with 15 °C at both room temperatures for both males and females (*p* = 0.001–0.038), except with a room temperature of 20 °C in females (*p* = 0.064).

The FSBPs at 30, 15, and 10 °C were lower in females than in males with both room temperatures (*p* < 0.001). However, the %FSBPs calculated at 15 and 10 °C did not differ between males and females (*p* = 0.38–0.71; Fig. 3).

The FSBPs within the male and female subgroups were not correlated with stature, weight, BMI, age, or finger volume (*p* = 0.33–0.52). The FSBPs at 30, 15, and 10 °C were not correlated with FST after the corresponding FSBP test within either males or females (*p* = 0.284–0.803). Similarly, there was no correlation between the %FSBPs calculated at either 15 or 10 °C and the FSTs within the males and females (*p* = 0.407–0.936).

### Association between baseline FSTs, finger rewarming times (*T*_4_ and *T*_2-base_), and %FSBP

The correlation coefficients between baseline finger skin temperatures, the time for the FST to increase by 4 °C (*T*_4_), the times for FST to return to within 2 °C of the baseline FST (*T*_2-base_), and the absolute FSBPs at 15 and 10 °C are shown in Table 3.

**Table 3:** Spearman rank correlation coefficients between baseline finger skin temperatures, times for FST to increase by 4 °C (*T*_4_), times for FST to return to within 2 °C of the baseline FST (*T*_2-base_), and absolute FSBP at 15 and 10 °C

	Room temperature of 20 °C			Room temperature of 28 °C		
	Baseline FST	*T*_4_	*T*_2-base_	Baseline FST	*T*_4_	*T*_2-base_
Finger rewarming						
Baseline FST	–	−0.626*	−0.571*	–	−0.295	−0.420
*T*_4_	−0.626*	–	0.638*	−0.295	–	0.504*
*T*_2-base_	−0.571*	0.638*	–	−0.420	0.504*	–

	Baseline FST	FSBP at 15 °C	FSBP at 10 °C	Baseline FST	FSBP at 15 °C	FSBP at 10 °C
Absolute finger systolic blood pressures, FSBP						
Baseline FST	–	0.783*	0.732*	–	0.577*	0.414
FSBP at 15 °C	0.783*	–	0.749*	0.577*	–	0.675*
FSBP at 10 °C	0.732*	0.749*	–	0.414	0.675*	–

Data from 24 subjects

*p < 0.05

With the room temperature at 20 °C, individual FSTs prior to cold provocation in the finger rewarming test were negatively correlated with *T*_4_ in both males and females (*p* = 0.013–0.034). However, with the room temperature at 28 °C, individual FSTs prior to cold provocation in the finger rewarming test were not correlated with *T*_4_ in either males or females (*p* = 0.069–0.115). A negative correlation was also found between baseline FSTs and *T*_2-base_ in both males and females at 20 °C (*p* = 0.008–0.027), but not at 28 °C (*p* = 0.119–0.338).

During the measurement of FSBPs at both room temperatures, the individual baseline FSTs were positively correlated with the absolute FSBPs obtained at 15 and 10 °C (*p* = 0.001–0.042), except for the FSBP at 10 °C with the room temperature of 28 °C in males (*p* = 0.137). There was no correlation between baseline FSTs and the %FSBP calculated at either 15 or 10 °C with either room temperature (*p* = 0.160–0.595).

The results suggest that healthy subjects with a higher baseline finger skin temperature tend to have quicker rewarming times and higher absolute FSBPs after cold provocation, but that these associations are dependent on the room temperature.

With both room temperatures, the times for FST to increase by 4 °C were negatively correlated with the %FSBP at 15 and 10 °C in both males and females (*p* = 0.006–0.039). A similar negative correlation was found between the times for FST to return to within 2 °C of the baseline FST and %FSBP at 15 and 10 °C (*p* = 0.012–0.024). This indicates that healthy subjects with higher percentage finger systolic blood pressures after digital cooling tend to have quicker recovery after immersion of their hands in 15 °C water for 5 min. There was no correlation between the absolute FSBP at 30 °C and the times for FST to increase by 4 °C or return to within 2 °C of the baseline FST (*p* = 0.125–0.847; Spearman).

## Discussion

### Finger skin temperature after cold provocation

In the current study, two indices have been used to indicate the rewarming times: the time for FST to increase by 4 °C and the time for FST to return to within 2 °C of the baseline FST. Over the 24 subjects, the median *T*_4_ was 162 s with the room temperature of 20 °C and 118 s with the room temperature of 28 °C, with the longest individual *T*_4_ of 314 s at 20 °C and 246 s at 28 °C. The median *T*_2-base_ was 21.5 min with the room temperature of 20 °C and 16.5 min with the room temperature of 28 °C, with the longest individual *T*_2-base_ of 27.4 min at 20 °C and 25.3 min at 28 °C. Evidently, there are large variations in individual finger rewarming times in healthy males and healthy females with both room temperatures.

The baseline finger skin temperature was dependent on room temperature, with higher finger temperatures when the room temperature was higher, consistent with expectations and previous studies (e.g. Mahbub et al. 2006; Mahbub and Harada 2008; Ye and Griffin 2011a). Finger skin temperature is indirectly related to finger blood flow and reflects the state of the capillaries and arteries. Mechanical, physiological, and pharmacological effects can therefore influence FST. Body core temperature is partially regulated by loss of heat via the skin, which depends on the difference between the temperature of the skin and the temperature of the environment. When the external environment is cold, or cooling, dermal blood vessels constrict causing the warm blood to bypass the skin and allow the skin temperature to drop towards the temperature of the environment.

Changes in FST after cold exposures depend on neurovascular processes that control the return to normal steady-state conditions. There are variations in the rate of rewarming with the 20 °C room temperature (Fig. 1), suggesting vasodilation during recovery is not mediated solely by gradual release of arterial vasospasm but through a combination of two or more processes (Lindsell and Griffin 2001).

Although using a different water temperature and a different immersion period, the dependence of the recovery of finger skin temperature on room temperature is consistent with Harada et al. (1998). An increase of 8 °C in the room temperature reduced by 44 s the time for the FST to increase by 4 °C and reduced by 5 min the time for the FST to return to within 2 °C of the baseline FST. Clearly, the room temperature must be well controlled when performing the test so as to obtain reliable times and compare them with a criterion for abnormal rewarming.

Finger skin temperatures on the contralateral hand not exposed to cold were reduced in both the FST test and the FSBP test. A reduced finger skin temperature contralateral to vibration stimulation has been reported in studies using both thermography (Nasu 1977) and thermocouples (Ye and Griffin 2011a). The presence of vasoconstriction in both hands after exposing one hand to a stressor (either cold or vibration) might be explained by reflex control of digital circulation mediated through central sympathetic activity (Fox and Edholm 1963; Roddie 1983). However, a study of FST on the contralateral hand in patients with the hand-arm vibration syndrome (HAVS) did not find significant changes with the same cold provocation conditions (Ye and Griffin 2016). The discrepancy might be due to the lower baseline FST (23.9–28.1 °C) in the HAVS patients or the dysfunction in the regulation of blood flow caused by their occupational exposures to vibration.

### Finger systolic blood pressures after cold provocation

Over the 24 subjects, the median %FSBP was 96.3% at 15 °C and 93.8% at 10 °C, with the lowest individual %FSBP of 79% at 15 °C and 74% at 10 °C. The median values are slightly higher than those in 22 male subjects aged 20–29 years where the median value was 94.8% at 15 °C and 90.3% at 10 °C, with the lowest individual %FSBP of 75% at 15 °C and 67% at 10 °C (Bovenzi 1988). The slightly greater reduction in FSBP with finger cooling from 30 to 15 or 10 °C in that study may due to different tobacco consumption within subjects, as smokers experienced a more intense digital vasoconstriction than non-smokers (Bovenzi 1988). This suggests that smoking history will influence the FSBP test and should be considered when making a medical decision.

When FSBPs were measured after digital cooling to 15 and 10 °C, higher FSBPs were found with a room temperature of 28 °C than 20 °C. However, there was a similar percentage change in FSBP at 15 and 10 °C relative to FSBP at 30 °C when corrected for any changes in FSBP in the thumb. The results confirm that room temperature influenced FSBP after cold provocation, but that by calculating the %FSBP the effect of room temperature is minimised. There is a similar trend with changes in finger blood flow before and during exposure to vibration at the same two room temperatures (i.e. 20 and 28 °C): reduced finger blood flow and smaller reductions in finger blood flow during exposure to vibration with a room temperature of 20 °C, but the percentage change in finger blood flow provoked by vibration is similar with both room temperatures (Ye and Griffin 2011a). Although the stimulus causing vasoconstriction was vibration not cold, the effect of room temperature on finger skin temperature, finger blood flow, and finger blood pressure seems broadly consistent.

### Gender effect

Lower baseline FSTs were found in females than in males, consistent with the previous studies suggesting increased sympathetic tone in females (Cooke et al. 1990; Ye and Griffin 2011b). The shorter rewarming times (*T*_4_ and *T*_2-base_) in males are consistent with a study suggesting that females show faster cooling and slower recovery in FST when in contact with cold solid materials (Jay and Havenith 2004). The skin differs in mechanical properties and thickness between males and females with the stratum corneum on the volar fingertips thinner in women than in men (Fruhstorfer et al. 2000). A difference between genders has also been reported in collagen and elastic fibre density, and in subjects aged 27–31 years the skin thickness is reported to be greater in males across the entire body, except for the lower back (Seidenari et al. 1994).

Males had higher finger blood pressures than females, both before and after the application of local cooling. Although the measurement location, measurement method, and environmental conditions differed, the findings are consistent with the previous studies (Wiinber et al. 1995; Khoury et al. 1992). In 352 normotensive (for age) Danish males and females aged 20–79 years, Wiinber et al. (1995) found that males had higher 24-h mean blood pressure, by approximately 6–10 mm Hg, than females, until the age of 70–79 years, when blood pressures were similar in males and females. A higher blood pressure among males has also been reported by Khoury et al. (1992) during ambulatory blood pressure monitoring on 131 males and females (aged 50–60 years).

Although the mechanisms responsible for gender differences in blood pressure are not clear, there is evidence that androgens, such as testosterone, play a role in gender-associated differences in blood pressure regulation. At ages 13–15 years, systolic blood pressure was approximately 4 mm Hg higher in boys than girls, and at 16–18 years, the systolic blood pressure was 10–14 mm Hg higher in boys (Harshfield et al. 1994). This suggested that after the onset of puberty, when androgen levels are increasing, blood pressure is higher in males than in females (Bachmann et al. 1987; Harshfield et al. 1994).

Body size may also contribute to differences in blood pressure between males and females. High body weight is linked with higher systolic and diastolic blood pressure (Seidman et al. 1991; Rosner et al. 2000). The body weight and body mass index were lower in the females of the current study but there were no significant correlations between finger systolic blood pressure and either body weight or body mass index. However, this might be explained by the small variations in body weight and body mass index in the subject groups.

### Diagnostic criteria

#### Finger skin temperature after cold provocation

One diagnostic indicator of abnormal finger rewarming is the time for the skin temperature on the distal phalanx of a finger to increase by 4 °C after cold provocation. If the time for a finger to rewarm by 4 °C is longer than 300 s, it is currently assumed to indicate ‘possible dysfunction’, whereas a rewarming time longer than 600 s is considered to indicate ‘possible dysfunction’ (Lindsell and Griffin 1998).

In this study with healthy subjects, the times for FSTs to increase by 4 °C were shorter than 300 s, except for one female subject with a *T*_4_ of 314 s with the room temperature at 20 °C. Using a *T*_4_ of 600 s as the threshold for distinguishing those with VWF from healthy individuals, none of the current subjects showed an abnormal reaction to cold.

Although a cold provocation test with one or both hand immersed in cold water is used worldwide, there is wide variation in the water temperature, the immersion time, and the parameters that are measured. International Standard 14835-1 (2016) recommends that when interpreting the rewarming data and assessing the validity of the data, information on the test conditions, subject conditions, temperature measurement, hand cooling, subject characteristics, and symptoms and signs observed during examination should be reported together with the measured values.

Several studies have been performed to compare different indices. Carnicelli et al. (1992) compared the repeatability of seven different parameters and found that the durations to increase by 3 and 4 °C exhibited the greatest repeatability. Based upon observations of 1,800 subjects examined with a cold provocation test, Gautherie et al. (1992) recommended the use of multiple indices of recovery: the delay in rewarming, the rate of recovery, and the recovery temperature compared to the initial temperature after 15 min.

The baseline FST, when measured in suitably controlled conditions, has been considered a useful indicator of peripheral vascular disorders not attributable to an exaggerated response to cold (Bovenzi 1987). In the current study, a negative correlation between baseline FST and rewarming times was only observed with a room temperature of 20 °C. The absence of an association between baseline FST and rewarming times with the room temperature of 28 °C may due to the narrow range of baseline FSTs within the two groups of subjects.

The time for FST to increase by 4 °C was significantly longer with the room temperature at 20 °C than 28 °C, and with females than males. This suggests that variations in the current diagnostic criterion for *T*_4_ may be required depending on the room temperature and gender. For example, it seems that a different set of normal values of *T*_4_ should be developed for females.

#### Finger systolic blood pressure after cold provocation

The diagnostic indicators of abnormal finger systolic blood pressure are the percentage changes in FSBP after cold provocation at 15 and 10 °C. A %FSBP lower than 80% is currently used to indicate ‘possible dysfunction’ and a %FSBP lower than 60% is considered to indicate ‘definite dysfunction’ (Lindsell and Griffin 1998).

In this study, all individual %FSBPs at 15 and 10 °C were greater than 80%, except for one female subject with %FSBPs of 79% and 74% at 15 and 10 °C, respectively. Using a %FSBP of 60% as a threshold for distinguishing patients with VWF from healthy individuals, none of the current healthy subjects showed an abnormal cold reaction.

Although the absolute FSBPs at 15 and 10 °C were lower with the room temperature of 20 °C (in females), the %FSBP calculated at 15 and 10 °C did not differ significantly between the two room temperatures, or between males and females. This suggests that using the calculated %FSBP as a diagnostic indicator, the variations in FSBP introduced by room temperature and gender can be minimised. This is consistent with a previous study that found the calculated %FSBP (corrected for changes in a reference finger) has a higher diagnostic accuracy than other finger systolic blood pressure indices (Bovenzi 2002). The findings also imply that if %FSBP is used as the diagnostic criterion, room temperature is not a major factor influencing the accuracy of the test when it is performed according to ISO 14835-2:2005. The tight control of room temperature (±1 °C) stated in ISO 14835-2:2005 seems unnecessary, allowing the test to be performed in environments where control of room temperature is difficult or impossible.

The study found that the time for fingers to rewarm by 4 °C is negatively correlated with %FSBPs at both 15 and 10 °C. This indicates that subjects show a similar trend in both tests: subjects with longer times to recover after cold provocation generally have lower FSBPs after local cooling. This is consistent with a comparison study of the two objective tests which showed a negative correlation between finger rewarming times and finger systolic blood pressures in hand-arm vibration syndrome patients with and without symptoms of finger whiteness (Ye and Griffin 2016). A review of objective tests for the diagnosis of VWF has concluded that both tests can provide a useful indication of dysfunction and assist the diagnosis of VWF (Mahbub and Harada 2011).

## Conclusions

Reductions in finger skin temperatures, finger rewarming times, and finger systolic blood pressures caused by local cooling are dependent on room temperature (20 or 28 °C). With a lower room temperature, pre-immersion finger skin temperatures are lower and the finger skin temperature takes longer to rewarm after immersion in cold water. Reductions in FSBP are also greater with a lower room temperature, but percentage changes in finger systolic blood pressure (i.e. %FSBP) are similar with 20 and 28 °C room temperatures. Females have lower finger skin temperatures, longer recovery periods, and lower finger systolic blood pressures before and after the application of local cooling of the hands or fingers, but percentage changes in finger systolic blood pressure (i.e. %FSBP) are similar in males and females.

The study shows that close control of room temperature (as required by ISO 14835-1:2016) is essential when measuring finger skin temperatures after cold provocation. However, close control of room temperature is not necessary when measuring percentage changes in finger systolic blood pressures after finger cooling. The criteria for ‘normal values’ are different for males and females when measuring finger rewarming times but similar when measuring percentage changes in finger systolic blood pressure (i.e. %FSBP).
